# Introducing activities of the Archives of Infectious Diseases History (AIDH) project: Historical epidemiology

**DOI:** 10.1186/s41182-020-00296-7

**Published:** 2021-01-27

**Authors:** Wataru Iijima, Hiroki Inoue, Tomoo Ichikawa

**Affiliations:** 1 Department of History, College of Literature, Aoyama Gakuin University, 4-4-25 Shibuya, Shibuya-ku, Tokyo, 150-8366 Japan; 2 Japan Society for the Promotion of Science, Kojimachi Business Center Building, 5-3-1 Kojimachi, Chiyoda-ku, Tokyo, 102-0083 Japan; 3 Department of Society and Regional Culture, Okinawa International University, 2-6-1 Ginowan, Okinawa, 901-2701 Japan

**Keywords:** The Archives of Infectious Diseases History (AIDH), Historical epidemiology, Historicalization, Oral interview, Historical materials

## Abstract

The AIDH is a project as a historical epidemiology. The AIDH aims to collect, maintain, and manage past epidemiological materials and to offer these materials to persons who are interested in the history and in the fields of tropical medicine and global health. In this paper, we introduce our purpose and activities and show a hypothesis about lymphatic filariasis with *Brugia malayi* in Japan as a case of historical epidemiology. We hope to build fruitful ties between historians and scholars of tropical medicine and global health workers through an interdisciplinary approach to the history of control of infectious diseases.

## Goal of the Archives of Infectious Diseases History (AIDH) project

During the last 10 years, we have launched the Archives of Infectious Diseases History (AIDH) project as an attempt at *historical epidemiology*: the collection of epidemiological materials outlining how to control a couple of infectious and parasitic diseases and the preparation of these materials for historians and persons who have great interest in the history of medicine and public health. By this project, we hope to provide a history of rich experiences related to the control of infectious and parasitic diseases in twentieth-century Japan.

This paper introduces our project, intended to present basic information related to the materials we are collecting and to present a role model of historical epidemiology. One goal of our project is to build fruitful ties between historians and scholars of tropical medicine and parasitic disease studies, and with global health workers using an interdisciplinary approach to the history of control of infectious and parasitic diseases in twentieth-century Japan.

Specifically, the AIDH project was launched to collect basic materials related to the elimination of infectious and parasitic diseases, such as malaria, schistosomiasis, lymphatic filariasis, and soil-transmitted helminths, and to offer these materials to persons interested in the history and the persons who devote great attention to the control of these diseases in the fields of tropical medicine and global health as academic and historical references. Through these new attempts, our project can be expected to contribute not only to the work of historians but also to that of many people who have great interest in the history of infectious and parasitic diseases. Furthermore, the AIDH project will ask many scholars in the fields of tropical medicine and parasitic disease studies to acknowledge the importance of historical epidemiology.

First, as one case of the AIDH project, we would like to discuss epidemiological materials related to malaria in Japan. From ancient times, vivax malaria and other types of malaria were prevalent throughout Japan, and many scholars who discussed malaria in the history of Japan described malaria patients using many descriptive materials, paintings, and other historical sources. Vivax was particularly prevalent on Honshu from ancient times. A couple of reports from the mid-nineteenth century describe the prevalence of vivax also in Hokkaido, the northern part of Japan, which has a cool climate. The prevalence of falciparum malaria was also noted in the sixteenth century for the Sakishima Islands, Yaeyama and Miyako, in southern Japan, a hot semi-tropical climate.

Anti-malaria campaigns were mounted in many districts in Japan around the twentieth century simultaneously with the development of tropical medicine and parasitic disease studies in Japan. It is noteworthy that Western-style tropical medicine and parasitic disease studies were imported from Germany in the latter nineteenth century by German scholars who taught in Japan as *Oyatoi-Gaikokujin*: foreign scholars and engineers employed by the Japanese government. Moreover, many Japanese students studied medicine and public health in Germany.

During this period, for instance, Erwin von Baelz (1849–1913) came from Leipzig, Germany, in 1876 and taught internal medicine at the Tokyo Medical College, the original medical school of The University of Tokyo, until his retirement in 1902. He also researched parasitic diseases in Japan such as trombiculiasis. Because his academic activities as *Oyatoi-Gaikokujin* were advanced in that age of the development of tropical medicine and parasitic disease studies in the Western world, he devoted much attention to parasitic diseases and his attempts at the discovery of new parasitic diseases in Japan. Because of strong German influence, the 30 years coinciding with Baelz’s work in Japan are known as the “German period” in the medical history of Japan [1].

Also based on the strong influence of German medicine in the late nineteenth century, the medical society of Japan established a Japanese style of Western medicine. Regarding the fields of parasitic disease studies, symbolic of the Japanization of Western medicine were the anti-malaria campaign on Taiwan under Japanese colonial rule, the discovery of *Schistosoma japonicum* by Fujiro Katsurada (1867–1946), and the study of *Oncomelania* snails, the intermediate host of *Schistosoma japonicum*, by Keinosuke Miyairi (1865–1946) and Minoru Suzuki (1885–1948) in the early twentieth century, as described later.

In the case of malaria, around the time of the Sino-Japanese War in the last decade of the nineteenth century, Japanese military forces faced severe prevalence of malaria in Taiwan. Vivax malaria and falciparum malaria had been prevalent from ancient times. They were among the main reasons that the Han Chinese did not migrate to Taiwan. After colonization, the Japanese colonial government on Taiwan devoted much attention to the control of malaria. Many Japanese scholars accumulated rich experience at treating and controlling malaria. From the first decade of the twentieth century, the Japanese colonial government launched an anti-malaria campaign by blood inspection and the distribution of quinine in areas where malaria was prevalent. Consequently, malaria control became a symbol of the legitimacy of the Japanese colonial rule and a channel for controlling the local and aboriginal societies in Taiwan. During the Japanese colonial rule, the anti-malaria campaign decreased the number of malaria patients moderately. Nevertheless, controlling malaria before WWII was difficult because the prevalence of malaria in Taiwan was based upon the development of agriculture and forestry. In the field of the social history of medicine, it is characterized as a “developogenic disease,” meaning that malaria was prevalent under the influence of agricultural development and urbanization.

The main method of controlling malaria in Taiwan under the Japanese colonial rule was quinine treatment administered to patients. Environmental efforts to control anopheles mosquitoes were also undertaken before WWII, but the budgets were limited. The Taiwan methods of blood inspection and quinine were emphasized in Taiwan under the Japanese colonial rule [2].

The history of malaria in Japan shows the interesting fact that Taiwan methods were introduced to Yaeyama and Miyako, both Okinawa Islands, because malaria was prevalent there just as it was in Taiwan. In the first half of the twentieth century, the Yaeyama local government started its anti-malaria campaign under the support of the central government in Tokyo and the Japanese colonial government on Taiwan. The main methods closely resembled those used in Taiwan: blood inspection and quinine were emphasized. The Yaeyama Public Health Bureau played an important role in the anti-malaria campaign: primary documents present details of malaria control before WWII. Moreover, many epidemiological materials are available to demonstrate the execution and outcomes of anti-malaria campaigns, including dichlorodiphenyltrichloroethane (DDT) spraying under the US military government after WWII. The authors were able to access these primary documents of the Okinawa Prefectural Archives and the Yaeyama Peace Memorial Museum.

Controlling vivax and falciparum malaria before the end of WWII in Yaeyama and Miyako was difficult, just as it was in Taiwan. Moreover, many people including primary school students in Hateruma of the Yaeyama Islands died of so-called War Malaria during their forced evacuation in 1945. After WWII, US military forces launched an anti-malaria campaign for Okinawa including Yaeyama and Miyako by means of vector control: mainly DDT spraying. Charles M. Wheeler, a medical officer of the 406th Medical General Laboratory, was a key person conducting the anti-malaria campaign in Okinawa. The primary sources described above show that mosquito control by DDT spraying was very effective. Local communities also played a very positive role in joining the anti-malaria campaign. Many primary documents describe anti-malaria measures in the 1950s in Yaeyama. The Okinawa Prefectural Archives and the Yaeyama Peace Memorial Museum include documents describing efforts advanced by the United States Civil Administration of the Ryukyus (USCAR) and the Ryukyu government after WWII. Combining materials of these two types, analyzing primary documents of the Yaeyama Public Health Bureau and primary documents of the USCAR and Ryukyu governments, one can reconstruct basic processes of malaria eradication in Yaeyama around the twentieth century [3].

Materials related to the anti-malaria campaign in Yaeyama, the Okinawa Islands, are interesting for historians striving to describe malaria control processes from a viewpoint of social history of medicine. They are also useful materials which devote attention to the control of malaria as a measure to advance tropical medicine and global health. The case of Okinawa is expected to be a good reference for their activities. In summary, rich experiences and primary documents available from Okinawa provide good lessons for many districts where malaria is still prevalent.

If one specifically examines the processes of infectious and parasitic disease control in twentieth-century Japan, then one finds a rich experience of control of malaria, schistosomiasis, lymphatic filariasis, and soil-transmitted helminth infections [4]. Moreover, the Japan Association of Parasite Control played an important role in the control of parasitic diseases of many kinds after WWII. These rich experiences were later exported and applied in Taiwan and Korea as medical aid efforts in the 1960s to the 1970s. Subsequently, the experiences of Japan were exported as rich experiences to South Asian countries through the JICA and some associations including Nihon Kiseichu Yobokai (the Japan Association of Parasite Control (JAPC)). Based upon these rich experiences, Global Parasite Control for the 21st Century, the so-called Hashimoto Initiative was declared by Prime Minister Ryutaro Hashimoto of Japan at the Birmingham Summit in 1998. He emphasized the importance of Japanese experiences because he thought that Japanese experiences can provide good lessons to control parasitic diseases in developing countries all over the world.

However, it is noteworthy that these basic materials, including primary documents accumulated through the process of anti-infectious and parasitic disease campaign, are facing hardship. They were almost spoiled after the control of diseases in Japan. Hikone, Shiga prefecture, was famous in Japanese history as a site of vivax malaria prevalence. After WWII, local governments and local residents devoted much attention to the control of malaria, led by many Japanese scholars and the US Army. From the US perspective, the control of famous local diseases such as malaria was a good exhibition of the legitimacy of the occupation of Japan. The local government launched an anti-malaria campaign with Japanese scholars who returned from Taiwan, such as Dr. Kaoru Morishita, and scholars of the US Army. Many local people joined the anti-malaria campaign. The case of Hikone provided a good lesson in the control of malaria. Unfortunately, the primary documents were almost destroyed, except for some few materials preserved by local governments and the US Army [5].

The AIDH project has devoted much attention to primary documents and basic materials accumulated during the process of controlling infectious and parasitic diseases such as malaria, schistosomiasis, lymphatic filariasis, and soil-transmitted helminth infections in the twentieth-century Japan. The main purposes of the AIDH project are to collect, maintain, and secure these primary documents and basic materials. They signal the “historicalization” of Japanese experiences for the control of infectious and parasitic diseases. In fact, their methods and activities can be regarded as historical epidemiology. Today, the project is collecting and managing many epidemiological and historical documents and materials. The following documents and materials are available now at the AIDH website [6].

## Data of historical epidemiology: the main AIDH project targets

### Materials of Prof. Yoshitaka Komiya: basic materials for schistosomiasis control

Prof. Yoshitaka Komiya (1900–1976) studied in the Faculty of Medicine School of Tokyo Imperial University and worked as a research associate at the Institute for Infectious Diseases of Tokyo Imperial University before WWII. Among his early research efforts, his main interest was social medicine under the solid influence of Dr. Teido Kunisaki (1894–1937), an associate professor of that Institute. It is noteworthy that Dr. Kunisaki had great sympathy for the communist movement. Dr. Komiya joined the political movement with him. As a result, Dr. Komiya was dismissed from the Institute for Infectious Diseases of Tokyo Imperial University. He began work at the Shanghai Science Institute in China, where he investigated a couple of infectious and parasitic diseases including schistosomiasis japonica in the Yangtze River Delta. Based on those efforts, Komiya changed his specialty to parasitology, including the study of lung flukes and schistosomiasis with the help of Chinese scholars such as Tao Jingsun (1897–1952).

After WWII, he returned to Japan and started to work at Maebashi Medical College. He devoted attention to the control of parasitic diseases based on his rich experiences in Shanghai. Later, he became the Director of the Department of Parasitology at the National Institute of Health (NIH), Tokyo. He played a key role in controlling schistosomiasis japonica in Yamanashi prefecture, which was famous for the prevalence of schistosomiasis japonica. His method, called the Yamanashi method or Komiya method, was an environmental change by concrete coverage of channels and control of *Oncomelania hupensis nosophora* [7].

In 1955, Dr. Manabu Sasa, a parasitologist and a professor of the Institute for Infectious Disease at The University of Tokyo, visited China as one member of a medical mission. To support the eradication of infectious and parasitic diseases in a severe pandemic area of schistosomiasis japonica, the Yangtze River Region, the Chinese Communist Party invited Japanese scholars including Prof. Sasa. Under a suggestion by Prof. Sasa, Prof. Komiya organized a research mission and visited China. In 1956, Prof. Komiya researched schistosomiasis japonica in many regions in China, including the Yangtze River Delta, with many Chinese scholars. He advised the Chinese government on controlling schistosomiasis japonica. On his advice, Prof. Komiya summarized Japanese experiences on methods of two types for anti-schistosomiasis programs: biochemical methods and the environmental methods. He emphasized the environmental method by reform of creeks and other small waterways using concrete [8]. Subsequently, the Chinese Communist Party put into operation an anti-schistosomiasis campaign in rural districts where this disease was prevalent.

Many materials including schistosomiasis control in Japan and China by Prof. Komiya were stored in the backyard at the National Institute of Infectious Diseases (National Institute of Health changed its name to the National Institute of Infectious Diseases). The AIDH project advanced the classification of many materials and prepared a catalog with a few scholars in the Department of Parasitology of the National Institute of Infectious Diseases (NIID). Some materials have since been moved to the Meguro Parasitological Museum, supported by Dr. Yasuyuki Morishima and Dr. Hiroshi Ohmae, the Department of Parasitology of NIID, and Dr. Kazuo Ogawa, the Director of the Meguro Parasitological Museum. Now, these materials are available to the public. The catalog is already available from our website. The collection comprises about 8000 articles, conference papers, and other materials presented in various languages.

### Materials of Prof. Masamitsu Otsuru

Masamitsu Otsuru (1919–2008) was born in Taiwan under Japanese colonial rule. He studied in the Faculty of Medicine of Taihoku Imperial University. He was interested in physical anthropology during his school age years. Although he worked as a research associate in the Faculty of Medicine for a very short time after graduation, the war-time regime changed his life. He was recruited as a medical officer of the Japanese Army and was stationed in Guangdong province in China at WWII. During his army life, his main work was to control malaria and other infectious diseases. Based on his army service experiences, he continued to research malaria and a couple of infectious diseases after returning to Japan. Otsuru became the first professor of parasitology in the Faculty of Medicine, Niigata University. There, he continued to research a couple of infectious and parasitic diseases including trombiculiasis. Moreover, he played an important role as an advisor of the Niigata Association of Parasite Control.

At the establishment of the Faculty of Medicine of the University of the Ryukyus, Prof. Otsuru moved to Naha from Niigata. He became the first dean of its faculty. His transfer to Okinawa was based on his Taiwan experience [9].

A couple of scholars had military experience as medical officers. They became leading scholars on infectious and parasitic diseases studies, as had Prof. Otsuru and others such as Prof. Manabu Sasa (Navy, Institute for Infectious Diseases, The University of Tokyo), Prof. Daisuke Katamine (Army, Research Institute of Endemics, Nagasaki University), and Prof. Muneo Yokogawa (Army, Chiba University). They were scholars born almost in identical years. The war-time regime recruited them as medical officers. They became leading scholars based on their military experience during WWII.

Professor Otsuru also had a chance to visit China in 1957. During his visit, he wrote a diary. A reprint has already been published. From his diary, one can confirm his rich activities in his research trip on parasitic diseases, malaria, schistosomiasis japonica, and lymphatic filariasis [10].

The AIDH project conducted research on the materials of Prof. Otsuru with the support of members of Otsuru Laboratory of the University of the Ryukyus; individuals including Prof. Yoshiya Sato, Dr. Hiroshi Toma, Dr. Daisuke Nonaka, and Ms. Kazue Kurashita; and many students of the University of the Ryukyus and the Graduate School of Aoyama Gakuin University.

Prof. Otsuru also took many photographs during his research work including China and other foreign countries because his hobby was photography. He liked to take photographs using his Leica camera. These photographs are also interesting materials, used at a time when many Japanese could not go abroad easily.

Now, many research materials produced by Prof. Otsuru on malaria and other infectious and parasitic diseases have already moved to the Meguro Parasitological Museum. A catalog will soon be available on our website. The collection comprises more than 1000 items including the records of research works, letters, photos, and many books based on his research interests. These materials are interesting and important for confirmation of the processes used to control infectious and parasitic diseases in post-war Japan. Furthermore, parts of materials having a close relation with Taiwan were moved to the Archives of the Institute of Taiwan History, Academia Sinica in Taipei. From these materials, we can confirm that Prof. Otsuru played an important role in controlling malaria and other parasitic diseases in Taiwan after WWII with many Taiwanese scholars who were good friends of the Faculty of Medicine of Taihoku Imperial University. Materials in the Archives of the Institute of Taiwan History have already been made available to the public. A catalog is available on the website now [11].

### Materials of Prof. Kaoru Morishita

Kaoru Morishita (1896–1978) studied at the Faculty of Science of the Tokyo Imperial University and researched malaria and mosquitoes in Taiwan under Japanese colonial rule. He worked at the Institute for Tropical Medicine of Taihoku Imperial University before WWII and continued to study at the Research Institute for Microbial Diseases at Osaka University after returning to Japan. He was the first president of the Japanese Society of Tropical Medicine. During the war-time period, he researched mosquitoes of New Guinea with the Japanese Navy. Malaria was one cause of death of Japanese soldiers both Army and Navy. It was important to conduct anti-malaria programs on and near the battlefields of China, Southeast Asia, and New Guinea [12].

We presume that his research materials at the laboratory of Osaka University were almost entirely destroyed, but we are nevertheless fortunate that some materials, such as the notebooks of mosquito research in New Guinea by Prof. Morishita, were kept at the laboratory of Prof. Otsuru. Upon the retirement of Prof. Morishita from Osaka University, some materials and many books were given to Prof. Otsuru at the University of Ryukyus. Actually, Prof. Otsuru offered many books belonging to Prof. Morishita to the Library of Medical College of the University of Ryukyus. Some materials described above were kept at the Otsuru Laboratory. The notebooks of Prof. Morishita kept at the Otsuru laboratory illustrate the history of tropical medicine in twentieth-century Japan. The materials of Prof. Morishita are now kept at Iijima Laboratory of the History Department of Aoyama Gakuin University, Tokyo. Now, the AIDH project is addressing methods to manage such materials with the Institute of Tropical Medicine, Nagasaki University, the Meguro Parasitological Museum, and the Toyo Bunko Library.

### Materials of Prof. Harujiro Kobayashi

Harujiro Kobayashi (1884–1969), a leading scholar of infectious and parasitic diseases studies and a contemporary of Prof. Morishita, worked at the Medical College of Keijo Imperial University. After WWII, Prof. Kobayashi continued to study at the Kyoto Prefectural University of Medicine [13]. A few materials related to his research work on parasitic diseases were preserved through the efforts of Prof. Yukio Yoshida and Dr. Minoru Yamada of the Department of Parasitic Diseases.

Materials belonging to Prof. Kobayashi are also kept in the Iijima Laboratory of the History Department of Aoyama Gakuin University, Tokyo. The AIDH also continues to discuss management of the materials with the Institute of Tropical Medicine, Nagasaki University, the Meguro Parasitological Museum, and the Toyo Bunko Library.

### Materials of the Institute of Tropical Medicine at Nagasaki University

In 1942, the Institute of Tropical Medicine was founded as the East Asia Research Institute of Endemic Disease (Toua Fudobyo Kenkyujo) at Nagasaki Medical University. After WWII, this institute re-launched research activities as the Research Institute of Endemic Disease (Fudobyo Kenkyujo) at Nagasaki University. For this new start, several scholars with experience in research of tropical medicines in colonial Taiwan played an important role.

During the 1950s and 1960s, the main research targets of this institute were lymphatic filariasis, schistosomiasis, and pulmonary distomiasis in Kyushu and the Ryukyu Islands. Around the end of the 1960s, they extended the achievements of both epidemiological research and medical cooperation in developing countries such as the Philippines (El Tor cholera and malaria) and Kenya (schistosomiasis). In 1967, the institute became the Institute of Tropical Medicine (Nettai Igaku Kenkyujo).

Collections of the Tropical Medicine Museum include publications by the institute (e.g., annual report, catalog of academic achievements), documents on field research, zoological specimens and experimental preparations, pictures, and videos (slides, VTR, and DVD).

The collection of the Department of Parasitology mainly comprises research documents, photographs, and films produced by Prof. Daisuke Katamine (1915–1991) during the 1950s and 1970s. The research topics were epidemiological research and mass drug administration (MDA) for lymphatic filariasis on the islands of Kyushu and Okinawa. This collection also includes collected books and research documents by Prof. Nansaburo Omori (1905–1988) between the 1950s and 1970s. Numerous library index and document boxes compiled by the department staff are worthy of special mention. This collection includes academic works of literature not only by Prof. Omori and his colleagues but also by specialists at other universities and institutes. For example, publications, photographs, and films of the Hikone Malaria Institute produced around 1950 are a valuable resource elucidating preventive measures for endemic diseases taken during the post-war period. A historical description of this topic has been published based on this collection [14].

### Materials of the Japan Association of Parasite Control

An important parasite control association is the Japan Association of Parasite Control (JAPC). The JAPC played a key role in parasitic disease control in Japan. In 1949, The Tokyo Parasite Control Association was established by Mr. Chojiro Kunii (1916–1996) and by Prof. Makoto Koizumi (1882–1952), a parasitologist. In 1955, the Japan Association of Parasite Control was founded as a voluntary association and a central organization of other similar local associations.

Under the leadership of Mr. Kunii, many parasitologists supported JAPC activities along with Prof. Yoshitaka Komiya and Prof. Kaoru Morishita, as well as central and local administrations and private foundations. Moreover, the JAPC actively involved local organizations such as women’s organizations. For example, the women’s organization of Sannomyo in Miyagi prefecture collaborated with the Miyagi Parasite Control Association, a Public Health Center, and teachers to held lectures on parasitic diseases and raised money through rice harvesting and purchasing projects and subsidized residents’ inspection and deworming expenses [15]. The women’s organization of Goka town in Ibaraki prefecture saved money and built improved toilets in about 75% of this area in the first year [16].

The JAPC’s main activities were fee-based fecal examinations and treatment at schools and in local societies, educational activities such as anti-parasite companies, organization of conferences, negotiations with administrators, and publications of journals, books, and pamphlets [17–19]. After the 1960s, the JAPC started overseas medical cooperation with foreign countries in the field of parasite control [20].

One important material related to the JAPC is the journal of the JAPC: *Kiseichu Yobo*. Its title means “Parasite Prevention.” *Kiseichu Yobo* was a monthly journal of the Tokyo Parasite Control Association and the JAPC published during 1949–1970. *Kiseichu Yobo* presents the JAPC activities in Japan and foreign countries. The AIDH project continues to collect JAPC materials and to support discussion of managing materials for the public.

## Interviews for oral history

How can we accumulate rich experiences related to the history of control of infectious and parasitic diseases? Oral interviews with persons who had conducted anti-infectious disease and parasitic disease campaign are a good method, constituting a so-called oral history.

Since 2015, the AIDH project has been carrying out oral interviews of more than 30 scholars, administrative staff members, nurses, and others involved with or affected by the prevalence of schistosomiasis, malaria, and lymphatic filariasis, including those of foreign countries. The AIDH will make some parts of these interviews available on its website. Records of these interviews are available at the Meguro Parasitological Museum.

### Prof. Somei Kojima

Somei Kojima (1940–) studied at the laboratory of Prof. Muneo Yokogawa, the School of Medicine, Chiba University. He worked at the School of Medicine of Chiba University and the Institute for Infectious Disease of The University of Tokyo. His oral interview was done in November 2015 on the main topic of his background in parasitic disease studies, research work on schistosomiasis, rich experiences of international health, and the “Hashimoto Initiative.”

### Prof. Isao Tada

Isao Tada (1936–) studied at the School of Medicine, Kyushu University, and subsequently worked at Medical Schools in Kagoshima, Kanazawa, and Kyushu. His oral interview was completed in December 2015. The main topic of his interview was his background of parasitic disease studies including lymphatic filariasis control in Cheju, Korea, experiences of the Japan–US Medical Cooperative Program, and activities in the field of international health in South America.

### Prof. Yoshiki Aoki

Yoshiki Aoki (1943–) studied at the Laboratory of Prof. Daisuke Katamine, the Research Institute of Endemics, Nagasaki University. He worked at the Institute of Tropical Medicine of Nagasaki University. His oral interview was done in January 2016, mainly on the topic of his background in parasitic disease studies including control of lymphatic filariasis in Cheju, Korea, and rich involvement in activities in support of international health in Africa based upon the Hashimoto Initiative.

### Prof. Tsutomu Takeuchi

Tsutomu Takeuchi (1945–2018) studied at the School of Medicine, Keio University. He worked at the same university. His oral interview was done in February 2016. The main topic of his interview was his background in parasitic disease studies and rich experience in international health and the Hashimoto Initiative.

### Dr. Takaaki Hara

Takaaki Hara (year of birth unknown) studied at the Faculty of Veterinary Medicine, Hokkaido University. He worked at the Japan Association of Parasite Control with Mr. Chojiro Kunii and worked at efforts to the control of parasitic diseases of many types. His interview was completed in April 2016. The topic was his background in activities with Kunii, rich experiences of school health, international health, and activities in the Hashimoto Initiative.

### Dr. Chokei Yoshida

Chokei Yoshida (1931–) graduated from the Department of Medicine, Nagoya University. He worked at the Laboratory of the Okinawa Public Health Bureau. His interview was done in July 2016. The topic of his interview was the background of his activities, anti-lymphatic filariasis campaign in Okinawa under the US occupation.

### Dr. Naoko Nihei

Naoko Nihei (1944–) graduated from Ochanomizu University as a scholar of medical geography. She studied at the Laboratory of the National Institute of Health and researched schistosomiasis from a viewpoint of medical geography. Her interview of June 2016 addressed the main topic of her rich experiences to control schistosomiasis in the Philippines and China and the field of international health. The Hashimoto Initiative was an important topic of her oral interview.

### Prof. Yukio Yoshida

Yukio Yoshida (year of birth unknown) graduated from the School of Medicine of the Kyoto Prefectural University of Medicine. He started his research work under the strong influence of Prof. Harujiro Kobayashi. His interview was conducted in July 2016 with Dr. Minoru Yamada. The main topic was the background of his parasitic disease studies and rich experiences in the control of parasitic diseases.

### Prof. Hiroshi Tanaka

Hiroshi Tanaka (year of birth unknown) graduated from the Faculty of Medicine of The University of Tokyo under the influence of Prof. Manabu Sasa. He later worked at the Institute for Infectious Disease of The University of Tokyo. The main topic of his interview that was completed in October 2016 was his rich experience in controlling lymphatic filariasis in Hachijo-koshima, Tokyo, and Amami, Kagoshima prefecture. Moreover, rich experiences in controlling schistosomiasis in the Philippines and the fields of international health in Africa were important topics.

### Mr. Fumio Kobayashi and Ms. Emiko Nakamura

Mr. Kobayashi (year of birth unknown) was an administrative staff member. Ms. Nakamura (year of birth unknown) was a nurse in some villages where lymphatic filariasis was prevalent in Ehime prefecture. The interview was conducted in August 2016. The main topic was the rich experience of the anti-lymphatic filariasis campaign in Misaki-cho, Ehime, under the strong influence of Prof. Manabu Sasa.

### Prof. Minggang Chen

Minggang Chen (1931–), an important scholar of parasitic disease studies in China, worked at the Institute for Parasitic Disease in Shanghai and conducted an anti-schistosomiasis campaign in many cities and rural districts near Shanghai. His interview was completed in September 2016 in Shanghai. The main topic of his interview was a rich experience in anti-schistosomiasis campaign activities in China and his WHO activities.

### Prof. Bayani L. Blas

Bayani L. Blas (year of birth unknown) was a key person who advanced the anti-schistosomiasis campaign in the Philippines, including the Leyte Islands. The main topic of the interview conducted in January 2016 in Tacloban, Leyte, was the anti-schistosomiasis campaign in Leyte Islands done with the staff of a US aids group and the activities with JICA, the Sasakawa Memorial Health Foundation. The JICA project devoted much attention to the control of schistosomiasis by vector control of the Yamanashi method. Subsequently, the Sasakawa Memorial Health Foundation emphasized the use of praziquantel, an effective drug for the treatment of schistosomiasis patients.

The JICA conducted medical aid activities including anti-schistosomiasis efforts, because many Filipinos died on the battlefields of the Asia-Pacific War. After support by the JICA, the anti-schistosomiasis campaign was continued by the Sasakawa Memorial Health Foundation. The Sasakawa Memorial Health Foundation kindly offered basic materials to the AIDH project.

## Why was it only in Hachijo-koshima: historical epidemiology of lymphatic filariasis with *Brugia malayi* in Japan

A mystery persists in the history of lymphatic filariasis in Japan. Why was *Brugia malayi*, which Prof. Manabu Sasa discovered, only prevalent in Hachijo-koshima [21]? The lymphatic filariasis had a long history in Japan. In fact, it was prevalent in Kagoshima, Nagasaki, Kochi, and Ehime prefectures. The Ryukyu-Okinawa islands also had prevalent lymphatic filariasis, especially in the Miyako islands. However, these cases involved *Wucheria bancrofti*.

In the history of the lymphatic filariasis in Japan, why *Brugia malayi* were only involved in Hachijo-koshima remains a mystery. Prof. Sasa and a couple of scholars of his laboratory, such as Dr. Shigeo Hayashi, examined the newly discovered *Brugia malayi*: it was a very special case in Japan. However, they did not explain the origin of *Brugia malayi* in Hachijo-koshima in their several research reports [22]. From an oral interview of Prof. Tanaka on *Brugia malayi* in Hachijo-koshima, it is very interesting and important that he pointed out that the origin of *Brugia malayi* was the migration of Ama divers from Cheju Islands of Korea. Based on his inference, *Brugia malayi* was imported by Korean Ama divers from the Cheju islands during the first half of the twentieth century. The Cheju Islands were a prevalent district of *Brugia malayi*, as were coastal districts of China, the Cho-shan Islands near Shanghai and Ninbo.

We have already checked government materials of the Tokyo Metropolitan Archives, but we were unable to confirm the prevalence of lymphatic filariasis *Brugia malayi* after WWII. A couple of materials describing the condition of infectious diseases and public health in Hachijo-koshima are available, but no information related to lymphatic filariasis *Brugia malayi* was forthcoming except in relation to tuberculosis and other infectious diseases [23]. Migration Labors including Ama diver from Cheju Islands to Japan at the twentieth century have been one of the main research topics in the field of history and sociology. Especially the Ama divers played an important role to collect gelidiaceae in many districts in Japan. The gelidiaceae was the material for traditional food, and it was also important as agricultural fertilizer. In Hachijo-koshima, they were also the main labor for the collection of gelidiaceae around WWII [24].

Research work on divers revealed that many divers in the Cheju Islands, 3928 male divers and 8373 female divers, and another 3360 divers went to Japan in 1936 [25]. Reports of governmental statistics show many divers in Japan in 1931: 40,538 divers comprising 18,763 male divers and 21,775 female divers. Among female divers, 8862 divers, or around 40%, had come from the Cheju Islands. One can infer that Ama divers from Cheju, *he-nyo* in the Korean language, constituted the majority group of divers in Japan [26].

According to an oral interview of Prof. Tanaka, Ama divers in Hachijo-koshima worked as collectors of gelidiaceae for food and agriculture. This background suggests that Ama divers from Cheju Islands imported *Brugia malayi* from the Cheju Islands in the twentieth century [27]. Prof. Sasa and Hayashi probably knew this relation between Japan and Korea, but they were afraid of criticism for racial discrimination, as speculated by Prof. Tanaka. Prof. Sasa and scholars of his laboratory did not broach these issues in field reports on *Brugia malayi* in Hachijo-koshima.

In the history of lymphatic filariasis in East Asia, it is very difficult to understand the basic situations on the spread of *Wucheria bancrofti* and *Brugia malayi*, because both two types of lymphatic filariasis were prevalent in Coastal China and the Korean peninsula. The vector was also a very important reason. At the control program by Prof. Sasa and the members of Tokyo University in 1950s, they also researched the mosquito types in the Hachijo-koshima and found out that the main vector of *Brugia malayi* was *Aedes togoi* [28]. It was very important that the main vector in Cheju Islands was also *Aedes togoi*. At the joint-control program for lymphatic filariasis in Cheju Islands around the 1970s between the Nagasaki University and the Seoul National University, both scholars also researched the mosquito species and found out the main vector of *Brugia malayi* was *Aedes togoi* [29]. This kind of analysis is not the direct evidence that the Ama divers were the reason that the *Brugia malayi* was only in Hachijo-koshima. But now we do not have the government materials and samples of lymphatic filariasis in Hachijo-koshima, the oral interview of Prof. Tanaka and the basic socio-economic situation of Hachijo-koshima including Ama diver from Cheju Islands has same suggestion on this issue.

From such analyses, we can confirm the importance of oral interviews. Now, the AIDH project has started joint research projects with Korean scholars on *Brugia malayi* in eastern Asia during the twentieth century.

## “I Shall Return” project: the Leyte schistosomiasis material repair program

After WWII, the US government and a couple of aid groups offered funds, staff, and new technologies to control schistosomiasis in the Philippines. After the withdrawal of US aid groups, the JICA organized an anti-schistosomiasis campaign in the Philippines including the Leyte Islands. The Schistosomiasis Control and Research Hospital was established through medical aid by the JICA to support anti-schistosomiasis campaigns based on Japan’s experiences including the Yamanashi method, as described previously. In the first stage of this program, Japanese staff members emphasized environmental methods as in Japan. However, efforts were not successful. Subsequently, the method was changed to praziquantel application by the support of the Sasakawa Memorial Foundation.

At the Schistosomiasis Control and Research Hospital, schistosomiasis patients received care. The Japanese staff members of the JICA conducted research work with Filipino scholars such as Prof. Blas. The AIDH project collected materials compiled by the Schistosomiasis Control and Research Hospital and also conducted oral interviews of the staff members of both Japan and the Philippines. However, in November 2013, a strong typhoon struck Leyte and destroyed basic materials in the Schistosomiasis Control and Research Hospital.

The “I Shall Return” project is a program for the repair of materials destroyed by the typhoon. Under the support of the Mitsubishi Foundation, the AIDH project started repairs with students of the History Department of Aoyama Gakuin University, Tokyo. After completion of the repairs, we returned the materials to the Leyte Islands. The basic situation related to the anti-schistosomiasis campaign and support by Japanese scholars, and the “I Shall Return” project is introduced at the Meguro Parasite Museum [30].

## Conclusion

The materials discussed in this paper and the materials of historical epidemiology are important for historians who are striving to reconstruct details of control of infectious and parasitic diseases in twentieth-century Japan. At the same time, materials of historical epidemiology can offer good references to a person who has conducted tropical medicine and global health because the processes of control of malaria, schistosomiasis, lymphatic filariasis, and soil-transmitted helminth infections are good lessons for districts in which these diseases are still prevalent.

Mass drug administration (MDA) is a commonly used method in the field of NTD control. Many cases of elimination of infectious diseases and parasitic diseases in Japan can demonstrate the importance of community-oriented methods. Inventing a community to control infectious and parasitic diseases and the level of intervention are controversial issues in these research fields. The cases in Japan and the Japanese experiences, as described earlier, are good references for scholars and practitioners in tropical medicine and parasitic disease studies.

To learn from rich experiences, we should collect, maintain, and manage various materials of epidemiology in the past. Now we start to refer to this our work as “historical epidemiology.” We should devote more attention to these activities with scholars of tropical medicine and parasitic disease studies. Therefore, the AIDH project represents a role model for future efforts.

## Data Availability

Not applicable.

## Abbreviations

AIDH: The Archives of Infectious Diseases History
USCAR: The United States Civil Administration of the Ryukyus
NIID: The National Institute of Infectious Diseases
MDA: Mass drug administration
JAPC: The Japan Association of Parasite Control
